# On Topological Properties for Benzenoid Planar Octahedron Networks

**DOI:** 10.3390/molecules27196366

**Published:** 2022-09-27

**Authors:** Jia-Bao Liu, Haidar Ali, Qurat Ul Ain, Parvez Ali, Syed Ajaz K. Kirmani

**Affiliations:** 1 School of Mathematics and Physics, Anhui Jianzhu University, Hefei 230601, China; 2 Department of Mathematics, Riphah International University, Faisalabad 38000, Pakistan; 3 Department of Mechanical Engineering, College of Engineering, Qassim University, Unaizah 56452, Saudi Arabia; 4 Department of Electrical Engineering, College of Engineering, Qassim University, Unaizah 56452, Saudi Arabia

**Keywords:** topological index, benzenoid planar octahedron networks, Randić index, forgotten index, reclassified Zagreb indices

## Abstract

Chemical descriptors are numeric numbers that capture the whole graph structure and comprise a basic chemical structure. As a topological descriptor, it correlates with certain physical aspects in addition to its chemical representation of underlying chemical substances. In the modelling and design of any chemical network, the graph is important. A number of chemical indices have been developed in theoretical chemistry, including the Wiener index, the Randić index, and many others. In this paper, we look at the benzenoid networks and calculate the exact topological indices based on the degrees of the end vertices.

## 1. Introduction

Topological indices, which are calculated using graph theory, are important tools. The chemical graph is a subbranch of graph theory with a wide range of applications in chemistry and mathematics. To expect the bioactivity of chemical substances, the topological indices, such as the ABC index, Wiener index, and Randić index, are very useful. A growing field called cheminformatics, which combines mathematics, information science, and chemistry, can be used to study quantitative structure–activity (QSAR) and structure–property (QSPR) relationships that are used to examine the organic activities and characteristics of biological substances. The topological index is a numerical value linked with chemical compositions that suggests a link between a variety of physical qualities and chemical structures that suggest a link between a variety of physical qualities, chemical reactivity, and biological activity. The translation of a chemical network into a number that describes the topology of the chemical network is the basis for topological indices. The topological index is a chemical descriptor that contains an integer associated with the graph that features the graph and does not change during graph automorphism. Interest in the computer chemistry area has already increased in terms of topological descriptors and is mostly related to the usage of uncommon quantities, the connection between structure properties, and the relationship between structure quantity. Some of the most common types of topological indices include those depending on distance, degree, and polynomials. Chemical graphs are commonly used to describe molecules and molecular compounds. A molecular graph provides a good example of the structural formula of chemical compounds in graph theory. Many researchers have recently discovered topological indices to be crucial in the analysis of the structural aspects of molecular graphs, networks, and chemical trees. A tree graph is an acyclic linked graph. The branch point of a tree is defined as any vertex with a degree of three or more.

## 2. Construction for Benzenoid Planar Octahedron Network BPOH(n)

The algorithm for constructing the benzenoid planar octahedron, benzenoid-dominating planar octahedron, and benzenoid hex planar octahedron networks (of dimension n) is as follows:

Step-1. We design an n-dimensional oxide network [1].
Step-2. After that, connect $C_{6}$ into each $C_{3}$ of the oxide network.
Step-3. The resultant graph is also known as the benzenoid planar octahedron network. Connect alternating adjacent $C_{6}$ vertices to each opposite vertex. The benzenoid-dominating planar octahedron network is represented by $B_{2}$, and the benzenoid hex planar octahedron network is represented by $B_{3}$. The benzenoid-dominating planar octahedron network BDPOH(n) and the benzenoid hex planar octahedron network BHPOH(n) can be created using this approach (n).

*B* is considered as a simple connected graph in this article, and the degree of each vertex s∈V(B) is denoted as δ(s).

Milan Randić [2] introduced the oldest, most desired, and most extensively examined degree-based topological index, which is known as the Randić index, denoted by $R_{-\frac{1}{2}}\left(B\right)$ and expressed as

(1) $$R_{-\frac{1}{2}}\left(B\right)=\sum_{st\in E(B)}\frac{1}{\sqrt{\delta (s)\delta (t)}}.$$

Furtula and Ivan Gutman [3] discovered the forgotten index, also known as the F-index, which is defined as

(2) $$F\left(B\right)=\sum_{st\in E(B)}\left({\delta (s)}^{2}+{\delta (t)}^{2}\right).$$

Balaban [4,5] discovered another important index, the Balaban index, in 1982. For a graph *B* with ‘*n*’ vertices and ‘*m*’ edges, the formula is

(3) $$J\left(B\right)=\left(\frac{m}{m-n+2}\right)\sum_{st\in E(B)}\frac{1}{\sqrt{\delta (s)\times \delta (t)}}.$$

Ranjini et al. [6] presented three types of reclassified Zagreb indices, which are defined as follows

(4) $$REZG_{1}\left(B\right)=\sum_{st\in E(B)}\left(\frac{\delta (s)\times \delta (t)}{\delta (s)+\delta (t)}\right).$$

(5) $$REZG_{2}\left(B\right)=\sum_{st\in E(B)}\left(\frac{\delta (s)+\delta (t)}{\delta (s)\times \delta (t)}\right).$$

(6) $$REZG_{3}\left(B\right)=\sum_{st\in E(B)}\left(\delta \left(s\right)\times \delta \left(t\right)\right)\left(\delta \left(s\right)+\delta \left(t\right)\right).$$

Only $ABC_{4}$ and $GA_{5}$ indices can be computed if we can determine the edge partition of these connectivity chemical networks consisting of the sum of the degree of the ending vertices of each edge in all of these graphs. $S_{s}=\sum_{v\in N_{B}\left(s\right)}deg\left(t\right)$ where $N_{G}\left(s\right)=\left\{t\in V\left(B\right)|st\in E\left(B\right)\right\}$.

Ghorbani et al. [7] introduced the $ABC_{4}$ index, which is described as

(7) $$ABC_{4}\left(B\right)=\sum_{st\in E(B)}\sqrt{\frac{S_{s}+S_{t}-2}{S_{s}.S_{t}}}.$$

Graovac et al. [8] introduced the fifth version of the GA index, which is as follows

(8) $$GA_{5}\left(B\right)=\sum_{st\in E(B)}\frac{2\sqrt{S_{s}S_{t}}}{S_{s}+S_{t}}.$$

## 3. Main Results

We compute the exact results for all of the above descriptors in this paper, such as $GA_{5}$. We suggest [9,10,11,12,13,14,15,16,17,18] for these results on various degree-based topological descriptors for a variety of graphs, and see [19,20] for basic notations and definitions.

### 3.1. Results for Benzenoid Planar Octahedron Network

We compute the *F*, *J*, $ReZG_{1}$, $ReZG_{2}$, $ReZG_{3}$, $ABC_{4}$, and $GA_{5}$ for indices for the benzenoid planar octahedron network denoted by $B_{1}\left(n\right)$ in this section.

**Theorem** **1.** *Consider the benzenoid planar octahedron network BPOH(n); its forgotten index is equal to*

$$F\left(BPOH\left(n\right)\right)=5580n^{2}-1152n.$$

**Figure 1:**
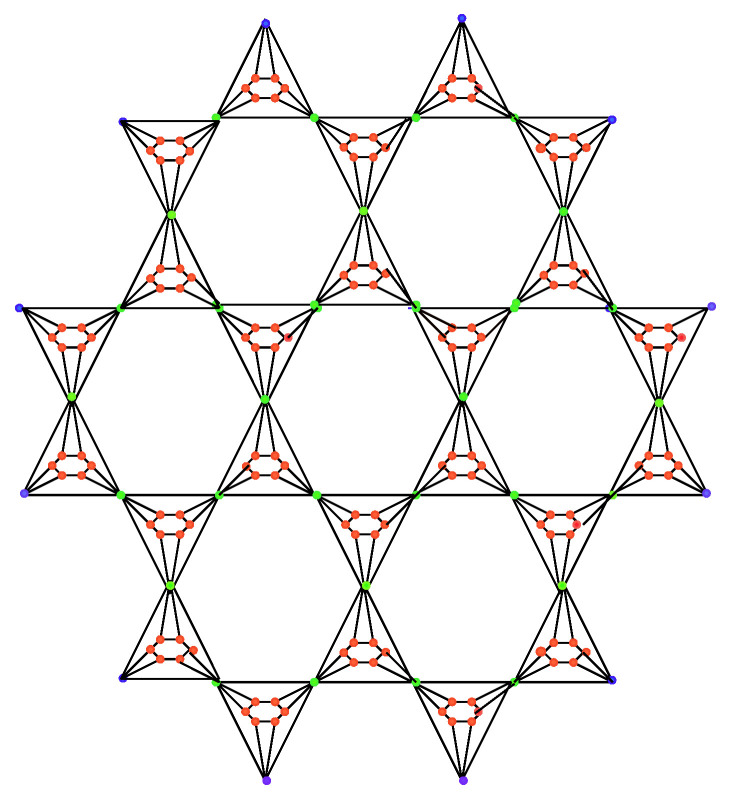
Benzenoid planar octahedron network BPOH(2).

**Proof.**  Let $B_{1}\left(n\right)$ be the benzenoid planar octahedron network BPOH(n), as shown in Figure 1, where n≥2 and $B_{1}\left(n\right)$ has $45n^{2}-3n$ vertices, and the edge set of $B_{1}\left(n\right)$ is divided into five partitions depending on the degrees of end vertices.

We can obtain the following result by using Table 1 edge partition, and using Equation (2), we have

$$\begin{array}{ccc} F(B_{1}\left(n\right)) & = & 18|E_{1}\left(B_{1}\left(n\right)\right)\left|+25\right|E_{2}\left(B_{1}\left(n\right)\right)\left|+73\right|E_{3}\left(B_{1}\left(n\right)\right)\left|+80\right|E_{4}\left(B_{1}\left(n\right)\right)|+\\ & & 128|E_{5}\left(B_{1}\left(n\right)\right)|, \end{array}$$

We obtain the following result after calculating it

$$⟹F\left(B_{1}\left(n\right)\right)=5580n^{2}-1152n.$$

□

In the following theorem, we compute Balaban index of benzenoid planar octahedron network BPOH(n).

**Theorem** **2.** *For the benzenoid planar octahedron network $B_{1}\left(n\right)$, the Balaban index is equal to*

$$J\left(B_{1}\left(n\right)\right)=\frac{45n^{3}\left(-6+6\sqrt{2}+8\sqrt{3}-4\sqrt{6}+3\left(19+4\sqrt{6}\right)\right)}{90n^{2}+6n+4}.$$

**Proof.**  Let $B_{2}\left(n\right)$ represent the benzenoid planar octahedron network. The outcomes can be obtained from Table 1 using the edge partition, and using Equation (3), we have

$$\begin{array}{ccc} J(B_{1}\left(n\right)) & = & \left(\frac{90n^{2}}{45n^{2}+3n+2}\right)(\frac{1}{3}|E_{1}\left(B_{1}\left(n\right)\right)|+\frac{1}{2\sqrt{3}}\left|E_{2}\left(B_{1}\left(n\right)\right)\right|+\\ & & \frac{1}{2\sqrt{6}}|E_{3}\left(B_{1}\left(n\right)\right)|+\frac{1}{4\sqrt{2}}|E_{4}\left(B_{1}\left(n\right)\right)|+\frac{1}{8}|E_{5}\left(B_{1}\left(n\right)\right)\left|\right), \end{array}$$

We obtain the following result by using, after calculating it,

$$⟹J\left(B_{1}\left(n\right)\right)=\frac{45n^{3}\left(-6+6\sqrt{2}+8\sqrt{3}-4\sqrt{6}+3\left(19+4\sqrt{6}\right)\right)}{90n^{2}+6n+4}.$$

□

**Theorem** **3.** *Let $B_{1}\left(n\right)$ be the benzenoid planar octahedron network n≥2. Then, we have*

$$\begin{array}{ccc} ReZG_{1}\left(B_{1}\left(n\right)\right) & = & \frac{2250}{11}n^{2}-\frac{1664}{77};\\ ReZG_{2}\left(B_{1}\left(n\right)\right) & = & 45n^{2}+3n;\\ ReZG_{3}\left(B_{1}\left(n\right)\right) & = & 29880n^{2}-9840n. \end{array}$$

**Proof.**  Let $B_{1}\left(n\right)$ represent the benzenoid planar octahedron network. The outcomes can be obtained from Table 1 using the edge partition, and using Equation (4), we have

$$\begin{array}{ccc} ReZG_{1}\left(B_{1}\left(n\right)\right) & = & \frac{3}{2}|E_{1}\left(B_{1}\left(n\right)\right)|+\frac{12}{7}|E_{2}\left(B_{1}\left(n\right)\right)|+\frac{24}{11}\left|E_{3}\left(B_{1}\left(n\right)\right)\right|\\ & & +\frac{8}{3}|E_{4}\left(B_{1}\left(n\right)\right)\left|+4\right|E_{5}\left(B_{1}\left(n\right)\right)|, \end{array}$$

We obtain the following result after calculating it

$$⟹ReZG_{1}\left(B_{1}\left(n\right)\right)=\frac{2250}{11}n^{2}-\frac{1664}{77}.$$

The $ReZG_{2}$ can be calculated by using (5) as follows

$$\begin{array}{ccc} ReZG_{2}\left(B_{1}\left(n\right)\right) & = & \frac{2}{3}|E_{1}\left(B_{1}\left(n\right)\right)|+\frac{7}{12}|E_{2}\left(B_{1}\left(n\right)\right)|+\frac{11}{24}\left|E_{3}\left(B_{1}\left(n\right)\right)\right|\\ & & +\frac{3}{8}|E_{4}\left(B_{1}\left(n\right)\right)|+\frac{1}{4}\left|E_{5}\left(B_{1}\left(n\right)\right)\right|, \end{array}$$

We obtain the following result after calculating it

$$⟹ReZG_{3}\left(B_{1}\left(n\right)\right)=45n^{2}+3n.$$

The $ReZG_{3}$ index can be calculated from (6) as follows

$$\begin{array}{ccc} ReZG_{3}\left(B_{1}\left(n\right)\right) & = & 54|E_{1}\left(B_{1}\left(n\right)\right)\left|+84\right|E_{2}\left(B_{1}\left(n\right)\right)\left|+264\right|E_{3}\left(B_{1}\left(n\right)\right)|\\ & & +384|E_{4}\left(B_{1}\left(n\right)\right)\left|+1024\right|E_{5}\left(B_{1}\left(n\right)\right)|, \end{array}$$

We obtain the following result after calculating it

$$⟹ReZG_{3}\left(B_{1}\left(n\right)\right)=29880n^{2}-9840n.$$

□

Now, we find $ABC_{4}$ and $GA_{5}$ indices of benzenoid planar octahedron network BPOH(n).

**Theorem** **4.** *Let $B_{1}\left(n\right)$ be the benzenoid planar octahedron network. Then:*

- *
$ABC_{4}\left(B_{1}\left(n\right)\right)=\frac{81\sqrt{43}}{55}n^{3}+\left(\frac{36}{\sqrt{11}}+\frac{18\sqrt{26}}{7}-\frac{162\sqrt{43}}{55}\right)n^{2}+(6\sqrt{\frac{3}{11}}+6\sqrt{\frac{6}{11}}+6\sqrt{\frac{22}{35}}+6\sqrt{\frac{41}{35}}-\;\frac{60}{\sqrt{11}}-\frac{3\sqrt{78}}{10}-\frac{9\sqrt{26}}{7}+\frac{81\sqrt{43}}{55})n+(-6\sqrt{\frac{3}{11}}+4\sqrt{\frac{7}{11}}-6\sqrt{\frac{41}{55}}+8\sqrt{\frac{6}{7}}+\sqrt{\frac{37}{5}}+\frac{24}{\sqrt{11}}-\frac{9\sqrt{78}}{20});$
*
- *
$GA_{5}\left(B_{1}\left(n\right)\right)=108n^{3}+\left(\frac{72\sqrt{154}}{29}-180\right)n^{2}+\left(102+2\sqrt{35}+3\sqrt{55}+\frac{16\sqrt{110}}{7}-\frac{120\sqrt{154}}{29}\right)n+\;(-18+\frac{72\sqrt{10}}{19}+\frac{144\sqrt{14}}{25}+\frac{72\sqrt{22}}{29}-\frac{3\sqrt{55}}{2}-\frac{16\sqrt{110}}{7}+\frac{48\sqrt{154}}{29}).$
*

**Proof.**  The $ABC_{4}\left(B_{1}\left(n\right)\right)$ can be calculated by using (7) as follows

$$\begin{array}{ccc} ABC_{4}\left(B_{1}\left(n\right)\right) & = & \frac{3\sqrt{2}}{10}|E_{1}\left(B_{1}\left(n\right)\right)|+\frac{\sqrt{770}}{70}|E_{2}\left(B_{1}\left(n\right)\right)|+\frac{\sqrt{26}}{14}\left|E_{3}\left(B_{1}\left(n\right)\right)\right|+\\ & & \frac{\sqrt{42}}{21}|E_{4}\left(B_{1}\left(n\right)\right)|+\frac{\sqrt{455}}{70}|E_{5}\left(B_{1}\left(n\right)\right)|+\frac{1}{\sqrt{11}}\left|E_{6}\left(B_{1}\left(n\right)\right)\right|\\ & & +\frac{\sqrt{66}}{22}|E_{7}\left(B_{1}\left(n\right)\right)|\frac{\sqrt{77}}{33}|E_{8}\left(B_{1}\left(n\right)\right)|+\frac{\sqrt{33}}{22}\left|E_{9}\left(B_{1}\left(n\right)\right)\right|+\\ & & \frac{\sqrt{185}}{60}|E_{10}\left(B_{1}\left(n\right)\right)|+\frac{\sqrt{78}}{40}|E_{11}\left(B_{1}\left(n\right)\right)|+\frac{\sqrt{2250}}{220}\left|E_{12}\left(B_{1}\left(n\right)\right)\right|\\ & & +\frac{\sqrt{86}}{44}\left|E_{13}\left(B_{1}\left(n\right)\right)\right|, \end{array}$$

We obtain the following result by using Table 2

$$\begin{array}{ccc} ABC_{4}\left(B_{1}\left(n\right)\right) & = & \frac{81\sqrt{43}}{55}n^{3}+\left(\frac{36}{\sqrt{11}}+\frac{18\sqrt{26}}{7}-\frac{162\sqrt{43}}{55}\right)n^{2}+(6\sqrt{\frac{3}{11}}\\ & & +6\sqrt{\frac{6}{11}}+6\sqrt{\frac{22}{35}}+6\sqrt{\frac{41}{35}}-\frac{60}{\sqrt{11}}-\frac{3\sqrt{78}}{10}-\frac{9\sqrt{26}}{7}+\frac{81\sqrt{43}}{55})n\\ & & +(-6\sqrt{\frac{3}{11}}+4\sqrt{\frac{7}{11}}-6\sqrt{\frac{41}{55}}+8\sqrt{\frac{6}{7}}+\sqrt{\frac{37}{5}}+\frac{24}{\sqrt{11}}-\frac{9\sqrt{78}}{20}). \end{array}$$

The index $GA_{5}$ can be determined from (7) as follows

$$\begin{array}{ccc} GA_{5}\left(B_{1}\left(n\right)\right) & = & |E_{1}\left(B_{1}\left(n\right)\right)|+\frac{\sqrt{35}}{6}|E_{2}\left(B_{1}\left(n\right)\right)\left|+\right|E_{3}(B_{1}\left(n))\right|\\ & & +\frac{6\sqrt{14}}{25}|E_{4}\left(B_{1}\left(n\right)\right)|+\frac{4\sqrt{35}}{27}|E_{5}\left(B_{1}\left(n\right)\right)|+\frac{2\sqrt{154}}{29}\left|E_{6}\left(B_{1}\left(n\right)\right)\right|\\ & & +\frac{\sqrt{55}}{8}|E_{7}\left(B_{1}\left(n\right)\right)|+\frac{6\sqrt{22}}{29}|E_{8}\left(B_{1}\left(n\right)\right)|+\frac{4\sqrt{55}}{31}\left|E_{9}\left(B_{1}\left(n\right)\right)\right|\\ & & +\frac{6\sqrt{10}}{19}|E_{10}\left(B_{1}\left(n\right)\right)\left|+\right|E_{11}\left(B_{1}\left(n\right)\right)|+\frac{2\sqrt{110}}{21}\left|E_{12}\left(B_{1}\left(n\right)\right)\right|\\ & & +|E_{13}\left(B_{1}\left(n\right)\right)|, \end{array}$$

We obtain the following result by using Table 2

$$\begin{array}{ccc} GA_{5}\left(B_{1}\left(n\right)\right) & = & 108n^{3}+\left(\frac{72\sqrt{154}}{29}-180\right)n^{2}+(102+2\sqrt{35}+3\sqrt{55}\\ & & +\frac{16\sqrt{110}}{7}-\frac{120\sqrt{154}}{29})n+(-18+\frac{72\sqrt{10}}{19}+\frac{144\sqrt{14}}{25}\\ & & +\frac{72\sqrt{22}}{29}-\frac{3\sqrt{55}}{2}-\frac{16\sqrt{110}}{7}+\frac{48\sqrt{154}}{29}). \end{array}$$

□

### 3.2. Results for Benzenoid-Dominating Planar Octahedron Network

We compute the *F*, *J*, $ReZG_{1}$, $ReZG_{2}$, $ReZG_{3}$, $ABC_{4}$, and $GA_{5}$ for indices for the benzenoid-dominating planar octahedron network denoted by $B_{2}\left(n\right)$ in this section.

**Theorem** **5.** *Consider the benzenoid-dominating planar octahedron network BDPOH(n); its forgotten index is equal to:*

$$F\left(BDPOH\left(n\right)\right)=16740n^{2}-19044n+6732.$$

**Figure 2:**
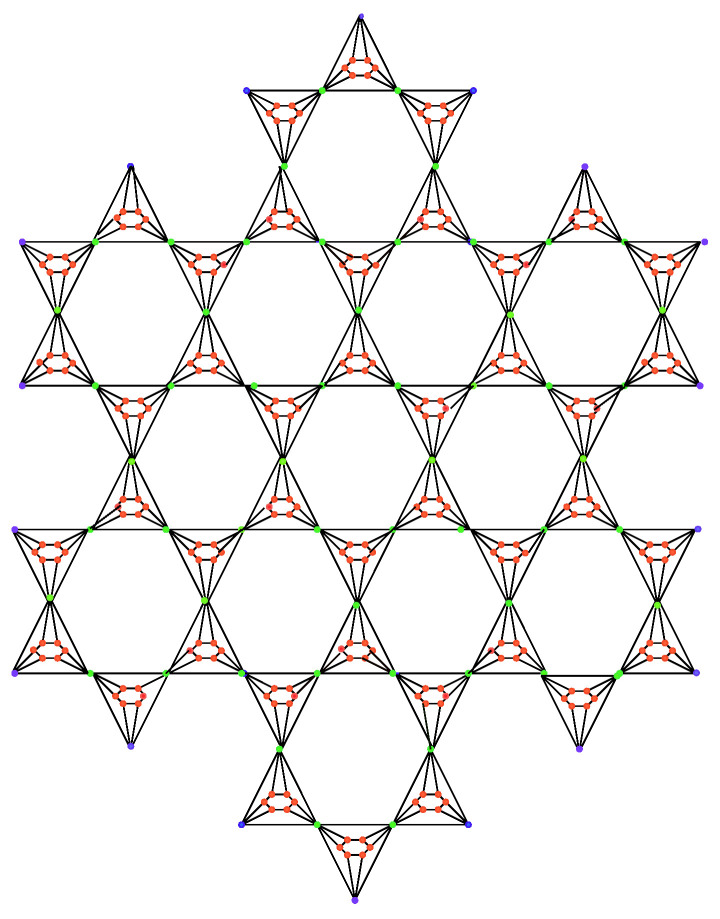
Benzenoid-dominating planar octahedron network BDPOH(2).

**Proof.**  Let $B_{2}\left(n\right)$ be the benzenoid-dominating planar octahedron network BDPOH(n), as shown in Figure 2, where n≥2 and $B_{2}\left(n\right)$ has $27n^{2}-33n+12$ vertices, and the edge set of $B_{2}\left(n\right)$ is divided into five partitions depending on the degrees of end vertices.

We can obtain the following result by using Table 3 edge partition, and using Equation (2), we have

$$\begin{array}{ccc} F_{1}\left(B_{2}\left(n\right)\right) & = & 18|E_{1}\left(B_{2}\left(n\right)\right)\left|+25\right|E_{2}\left(B_{2}\left(n\right)\right)\left|+73\right|E_{3}\left(B_{2}\left(n\right)\right)|\\ & & +80|E_{4}\left(B_{2}\left(n\right)\right)\left|+128\right|E_{5}\left(B_{2}\left(n\right)\right)|, \end{array}$$

We obtain the following result after calculating it

$$⟹F\left(B_{2}\left(n\right)\right)=16740n^{2}-19044n+6732.$$

□

**Table 3:** Degree-Based Edge Partition for BDPOH(n).

(δ(s),δ(t))	Number of Edges	(δ(s),δ(t))	Number of Edges
(3,3)	$108n^{2}-132n+48$	(4,8)	24n−12
(3,4)	24n−12	(8,8)	$54n^{2}-162n+84$
(3,8)	$108n^{2}-132n+48$		

In the following theorem, we compute Balaban index of benzenoid-dominating planar octahedron network BDPOH(n).

**Theorem** **6.** *For the benzenoid-dominating planar octahedron network $B_{2}\left(n\right)$, the Balaban index is equal to:*

$$\begin{array}{c} J\left(B_{2}\left(n\right)\right)=\frac{1}{4}(\frac{18\sqrt{2}\left(2n-1\right)\left(45n^{2}-43n+14\right)}{135n^{2}-141n+50}+4\sqrt{6}\left(9n^{2}-11n+4\right)\\ +3\left(9n^{2}-13n+5\right)+48\left(3n^{2}-3n+1\right)+8\sqrt{3}\left(-1+2n\right)). \end{array}$$

**Proof.**  Let $B_{2}\left(n\right)$ represent the benzenoid-dominating planar octahedron network. The outcomes can be obtained from Table 3 using the edge partition, and using Equation (3), we have

$$\begin{array}{ccc} J(B_{2}\left(n\right)) & = & \left(\frac{90n^{2}}{45n^{2}+3n+2}\right)(\frac{1}{3}|E_{1}\left(B_{2}\left(n\right)\right)|+\frac{1}{2\sqrt{3}}|E_{2}\left(B_{2}\left(n\right)\right)|+\frac{1}{2\sqrt{6}}\left|E_{3}\left(B_{2}\left(n\right)\right)\right|\\ & & +\frac{1}{4\sqrt{2}}|E_{4}\left(B_{2}\left(n\right)\right)|+\frac{1}{8}|E_{5}\left(B_{2}\left(n\right)\right)\left|\right), \end{array}$$

We obtain following result by performing calculation

$$\begin{array}{c} ⟹J(B_{2}\left(n\right))=\frac{1}{4}(\frac{18\sqrt{2}\left(2n-1\right)\left(45n^{2}-43n+14\right)}{135n^{2}-141n+50}+4\sqrt{6}\left(9n^{2}-11n+4\right)\\ +3\left(9n^{2}-13n+5\right)+48\left(3n^{2}-3n+1\right)+8\sqrt{3}\left(-1+2n\right)). \end{array}$$

□

**Theorem** **7.** *Let $B_{2}\left(n\right)$ be the benzenoid-dominating planar octahedron network n≥2. Then, we have*

$$\begin{array}{ccc} ReZG_{1}\left(B_{2}\left(n\right)\right) & = & \frac{6750}{11}n^{2}-\frac{4598}{7}n+\frac{17414}{77};\\ ReZG_{2}\left(B_{2}\left(n\right)\right) & = & 135n^{2}-129n+42;\\ ReZG_{3}\left(B_{2}\left(n\right)\right) & = & 89640n^{2}-110160n+40140. \end{array}$$

**Proof.**  Let $B_{2}\left(n\right)$ represent the benzenoid-dominating planar octahedron network. The outcomes can be obtained from Table 3, and using the edge partition as follows and using Equation (4), we have

$$\begin{array}{ccc} ReZG_{1}\left(B_{2}\left(n\right)\right) & = & \frac{3}{2}|E_{1}\left(B_{2}\left(n\right)\right)|+\frac{12}{7}|E_{2}\left(B_{2}\left(n\right)\right)|+\frac{24}{11}\left|E_{3}\left(B_{2}\left(n\right)\right)\right|\\ & & +\frac{8}{3}|E_{4}\left(B_{2}\left(n\right)\right)\left|+4\right|E_{5}\left(B_{2}\left(n\right)\right)|, \end{array}$$

We obtain the following result after calculating it

$$ReZG_{1}\left(B_{2}\left(n\right)\right)=\frac{6750}{11}n^{2}-\frac{4598}{7}n+\frac{17414}{77}.$$

The $ReZG_{2}$ can be calculated by using (5) as follows

$$\begin{array}{ccc} ReZG_{2}\left(B_{2}\left(n\right)\right) & = & \frac{2}{3}|E_{1}\left(B_{2}\left(n\right)\right)|+\frac{7}{12}|E_{2}\left(B_{2}\left(n\right)\right)|+\frac{11}{24}\left|E_{3}\left(B_{2}\left(n\right)\right)\right|\\ & & +\frac{3}{8}|E_{4}\left(B_{2}\left(n\right)\right)|+\frac{1}{4}\left|E_{5}\left(B_{2}\left(n\right)\right)\right|, \end{array}$$

We obtain the following result after calculating it

$$ReZG_{2}\left(B_{2}\left(n\right)\right)=135n^{2}-129n+42.$$

The $ReZG_{3}$ index can be calculated from (6) as follows

$$\begin{array}{ccc} ReZG_{3}\left(B_{2}\left(n\right)\right) & = & 54|E_{1}\left(B_{2}\left(n\right)\right)\left|+84\right|E_{2}\left(B_{2}\left(n\right)\right)\left|+264\right|E_{3}\left(B_{2}\left(n\right)\right)|\\ & & +384|E_{4}\left(B_{2}\left(n\right)\right)\left|+1024\right|E_{5}\left(B_{2}\left(n\right)\right)|, \end{array}$$

We obtain the following result after calculating it

$$⟹ReZG_{3}\left(B_{2}\left(n\right)\right)=89640n^{2}-110160n+40140.$$

□

Now, we find $ABC_{4}$ and $GA_{5}$ indices of benzenoid-dominating planar octahedron network BDPOH(n).

**Theorem** **8.** *Let $B_{2}\left(n\right)$ be the benzenoid-dominating planar octahedron network. Then:*

- *
$ABC_{4}(B_{2}\left(n\right))=\frac{486\sqrt{43}}{55}n^{3}+\left(\frac{114}{\sqrt{11}}+\frac{51\sqrt{26}}{7}-\frac{1296\sqrt{43}}{11}\right)n^{2}+\left(6\sqrt{\frac{2}{3}}+12\sqrt{\frac{13}{35}}+12\sqrt{\frac{6}{11}}+\;12\sqrt{\frac{22}{35}}+4\sqrt{\frac{7}{11}}+6\sqrt{\frac{41}{55}}+8\sqrt{\frac{6}{7}}+\sqrt{\frac{37}{5}}-\frac{210}{\sqrt{11}}-\frac{57\sqrt{26}}{7}+\frac{567\sqrt{43}}{55}\right)+(-6\sqrt{\frac{3}{11}}-6\sqrt{\frac{6}{11}}-\;6\sqrt{\frac{22}{35}}-6\sqrt{\frac{41}{55}}-\sqrt{\frac{37}{5}}+\frac{84}{\sqrt{11}}+\frac{\sqrt{70}}{6}+\frac{9\sqrt{26}}{7}-\frac{27\sqrt{43}}{11};$
*
- *
$GA_{5}(B_{2}\left(n\right))=648n^{3}+\left(-1590+\frac{288\sqrt{154}}{29}\right)n^{2}+\left(1290+\frac{72\sqrt{10}}{19}+\frac{144\sqrt{14}}{25}+\frac{72\sqrt{22}}{29}+\frac{68\sqrt{35}}{9}+\;\frac{141\sqrt{55}}{31}+\frac{16\sqrt{110}}{7}-\frac{420\sqrt{154}}{29}\right)n+(336-\frac{72\sqrt{10}}{19}-2\sqrt{35}-\frac{189\sqrt{55}}{62}-\frac{16\sqrt{110}}{7}+\frac{168\sqrt{154}}{29}.$
*

**Proof.**  Using the edge partition, we have

$$\begin{array}{ccc} ABC_{4}\left(B_{2}\left(n\right)\right) & = & \frac{3\sqrt{2}}{10}|E_{1}\left(B_{2}\left(n\right)\right)|+\frac{\sqrt{770}}{70}|E_{2}\left(B_{2}\left(n\right)\right)|+\frac{\sqrt{26}}{14}\left|E_{3}\left(B_{2}\left(n\right)\right)\right|\\ & & +\frac{\sqrt{42}}{21}|E_{4}\left(B_{2}\left(n\right)\right)|+\frac{\sqrt{455}}{70}|E_{5}\left(B_{2}\left(n\right)\right)|+\frac{1}{\sqrt{11}}\left|E_{6}\left(B_{2}\left(n\right)\right)\right|\\ & & +\frac{\sqrt{66}}{22}|E_{7}\left(B_{2}\left(n\right)\right)|+\frac{\sqrt{77}}{33}|E_{8}\left(B_{2}\left(n\right)\right)|+\frac{\sqrt{33}}{22}\left|E_{9}\left(B_{2}\left(n\right)\right)\right|\\ & & +\frac{\sqrt{185}}{60}|E_{10}\left(B_{2}\left(n\right)\right)|+\frac{\sqrt{78}}{40}|E_{11}\left(B_{2}\left(n\right)\right)|+\frac{\sqrt{2250}}{220}\left|E_{12}\left(B_{2}\left(n\right)\right)\right|\\ & & +\frac{\sqrt{86}}{44}\left|E_{13}\left(B_{2}\left(n\right)\right)\right|, \end{array}$$

We obtain the following result by using Table 4

$$\begin{array}{ccc} ABC_{4}\left(B_{2}\left(n\right)\right) & = & \frac{486\sqrt{43}}{55}n^{3}+\left(\frac{114}{\sqrt{11}}+\frac{51\sqrt{26}}{7}-\frac{1296\sqrt{43}}{11}\right)n^{2}+(6\sqrt{\frac{3}{11}}\\ & & +12\sqrt{\frac{13}{35}}+12\sqrt{\frac{6}{11}}+12\sqrt{\frac{22}{35}}+4\sqrt{\frac{7}{11}}+6\sqrt{\frac{41}{55}}+8\sqrt{\frac{6}{7}}+\\ & & \sqrt{\frac{37}{5}}-\frac{210}{\sqrt{11}}-\frac{57\sqrt{26}}{7}+\frac{567\sqrt{43}}{55})n+(-6\sqrt{\frac{3}{11}}-6\sqrt{\frac{6}{11}}\\ & & -6\sqrt{\frac{22}{35}}-6\sqrt{\frac{41}{55}}-\sqrt{\frac{37}{5}}+\frac{84}{\sqrt{11}}+\frac{\sqrt{70}}{6}+\frac{9\sqrt{26}}{7}-\frac{27\sqrt{43}}{11}). \end{array}$$

The index $GA_{5}$ can be determined from (8) as follow

$$\begin{array}{ccc} GA_{5}\left(B_{2}\left(n\right)\right) & = & |E_{1}\left(B_{2}\left(n\right)\right)|+\frac{\sqrt{35}}{6}|E_{2}\left(B_{2}\left(n\right)\right)\left|+\right|E_{3}\left(B_{2}\left(n\right)\right)|+\frac{6\sqrt{14}}{25}\left|E_{4}\left(B_{2}\left(n\right)\right)\right|\\ & & +\frac{4\sqrt{35}}{27}|E_{5}\left(B_{2}\left(n\right)\right)|+\frac{2\sqrt{154}}{29}|E_{6}\left(B_{2}\left(n\right)\right)|+\frac{\sqrt{55}}{8}\left|E_{7}\left(B_{2}\left(n\right)\right)\right|+\\ & & \frac{6\sqrt{22}}{29}|E_{8}\left(B_{2}\left(n\right)\right)|+\frac{4\sqrt{55}}{31}|E_{9}\left(B_{2}\left(n\right)\right)|+\frac{6\sqrt{10}}{19}\left|E_{10}\left(B_{2}\left(n\right)\right)\right|\\ & & +|E_{11}\left(B_{2}\left(n\right)\right)|+\frac{2\sqrt{110}}{21}|E_{12}\left(B_{2}\left(n\right)\right)\left|+\right|E_{13}\left(B_{2}\left(n\right)\right)|, \end{array}$$

We obtain the following result using Table 4

$$\begin{array}{ccc} GA_{5}\left(B_{2}\left(n\right)\right) & = & 648n^{3}+\left(-1590+\frac{288\sqrt{154}}{29}\right)n^{2}+(1290+\frac{72\sqrt{10}}{19}+\frac{144\sqrt{14}}{25}\\ & & +\frac{72\sqrt{22}}{29}+\frac{68\sqrt{35}}{9}+\frac{141\sqrt{55}}{31}+\frac{16\sqrt{110}}{7}-\frac{420\sqrt{154}}{29})n+\\ & & \left(336-\frac{72\sqrt{10}}{19}-2\sqrt{35}-\frac{189\sqrt{55}}{62}-\frac{16\sqrt{110}}{7}+\frac{168\sqrt{154}}{29}\right) \end{array}$$

□

### 3.3. Results for Benzenoid Hex Planar Octahedron Network

In this section, we compute certain degree-based topological indices of benzenoid hex planar octahedron network denoted by $B_{3}\left(n\right)$ and compute the *F*, *J*, $ReZG_{1}$, $ReZG_{2}$, $ReZG_{3}$, $ABC_{4}$, and $GA_{5}$ indices for benzenoid hex planar octahedron network in this section.

**Theorem** **9.** *Consider the benzenoid hex planar octahedron network BHPOH(n); its forgotten index is equal to:*

$$F\left(BHPOH\left(n\right)\right)=5580n^{2}+4008n+48.$$

**Figure 3:**
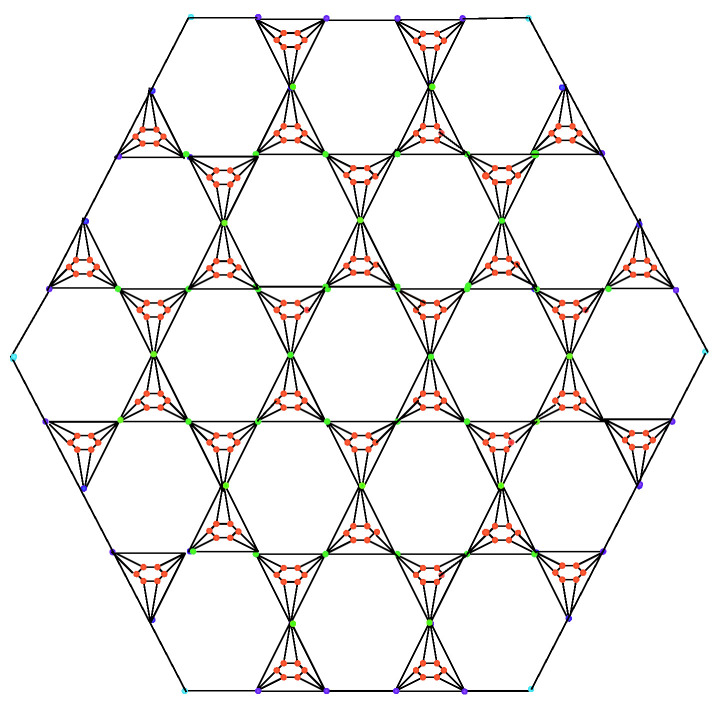
Benzenoid hex planar octahedron network BHPOH(2).

**Proof.**  Let $B_{3}\left(n\right)$ be the benzenoid hex planar octahedron network BHPOH(n) as shown in Figure 3, where n≥2 and $B_{3}\left(n\right)$ has $45n^{2}+51n+6$ vertices and the edge set of $B_{3}\left(n\right)$ is divided into seven partitions based on the degrees of end vertices.

We can obtain the following result by using Table 5 edge partition.

$$\begin{array}{ccc} F_{1}\left(B_{3}\left(n\right)\right) & = & 29|E_{1}\left(B_{3}\left(n\right)\right)\left|+18\right|E_{2}\left(B_{3}\left(n\right)\right)\left|+34\right|E_{3}\left(B_{3}\left(n\right)\right)\left|+128\right|E_{4}\left(B_{3}\left(n\right)\right)|+\\ & & 73|E_{5}\left(B_{3}\left(n\right)\right)\left|+50\right|E_{6}\left(B_{3}\left(n\right)\right)\left|+89\right|E_{7}\left(B_{3}\left(n\right)\right)|, \end{array}$$

We obtain the following result after calculating it

$$⟹F\left(B_{3}\left(n\right)\right)=5580n^{2}+4008n+48.$$

□

**Table 5:** Degree-based edge partition for BHPOH(n).

(δ(s),δ(t))	Number of Edges	(δ(s),δ(t))	Number of Edges
(2,5)	12	(5,5)	12n−6
(3,3)	$36n^{2}-36n$	(5,8)	12n
(3,5)	24n	(8,8)	$18n^{2}$
(3,8)	$36n^{2}+12n$		

In the following theorem, we compute Balaban index of benzenoid hex planar octahedron network BHPOH(n).

**Theorem** **10.** *For the benzenoid hex planar octahedron network $B_{3}\left(n\right)$, the Balaban index is equal to:*

$$\begin{array}{ccc} J(B_{3}\left(n\right)) & = & \frac{1}{10(45n^{2}+45n+2)}3\left(15n^{2}+16n+1\right)(24\left(-1+\sqrt{10}\right)\\ & & +4\left(72+5\sqrt{6}+3\sqrt{10}+8\sqrt{15}\right)n+15\left(19+4\sqrt{6}\right)n^{2}). \end{array}$$

**Proof.**  Let $B_{3}\left(n\right)$ represent the benzenoid hex planar octahedron network. The outcomes can be obtained from Table 5 using the edge partition, and using Equation (3), we have

$$\begin{array}{ccc} J(B_{3}\left(n\right)) & = & \frac{90n^{2}+96n+6}{45n^{2}+45n+2}(\frac{1}{\sqrt{10}}|E_{1}\left(B_{3}\left(n\right)\right)|+\frac{1}{3}\left|E_{2}\left(B_{3}\left(n\right)\right)\right|\\ & & +\frac{1}{\sqrt{15}}|E_{3}\left(B_{3}\left(n\right)\right)|+\frac{1}{2\sqrt{6}}|E_{4}\left(B_{3}\left(n\right)\right)|+\frac{1}{5}\left|E_{5}\left(B_{3}\left(n\right)\right)\right|\\ & & +\frac{1}{2\sqrt{10}}|E_{6}\left(B_{3}\left(n\right)\right)|+\frac{1}{8}|E_{7}\left(B_{3}\left(n\right)\right)\left|\right), \end{array}$$

We obtain the following result after calculating it

$$\begin{array}{ccc} ⟹J(B_{3}\left(n\right)) & = & \frac{1}{10(45n^{2}+45n+2)}3\left(15n^{2}+16n+1\right)(24\left(-1+\sqrt{10}\right)\\ & & +4\left(72+5\sqrt{6}+3\sqrt{10}+8\sqrt{15}\right)n+15\left(19+4\sqrt{6}\right)n^{2}). \end{array}$$

□

**Theorem** **11.** *Let $B_{3}\left(n\right)$ be the benzenoid hex planar octahedron network n≥2. Then, we have*

$$\begin{array}{ccc} ReZG_{1}\left(B_{3}\left(n\right)\right) & = & \frac{2250}{11}n^{2}+\frac{27471}{143}n+\frac{15}{7};\\ ReZG_{2}\left(B_{3}\left(n\right)\right) & = & 45n^{2}+51n+6;\\ ReZG_{3}\left(B_{3}\left(n\right)\right) & = & 29880n^{2}+17232-660. \end{array}$$

**Proof.**  Let $B_{3}\left(n\right)$ represent the benzenoid hex planar octahedron network. The outcomes can be obtained from Table 5 using the edge partition, and using Equation (4), we have

$$\begin{array}{ccc} ReZG_{1}\left(B_{3}\left(n\right)\right) & = & \frac{10}{7}|E_{1}\left(B_{3}\left(n\right)\right)|+\frac{3}{2}|E_{2}\left(B_{3}\left(n\right)\right)|+\frac{15}{8}\left|E_{3}\left(B_{3}\left(n\right)\right)\right|\\ & & +\frac{24}{11}|E_{4}\left(B_{3}\left(n\right)\right)|+\frac{5}{2}|E_{5}\left(B_{3}\left(n\right)\right)|+\frac{40}{13}\left|E_{6}\left(B_{3}\left(n\right)\right)\right|\\ & & +4|E_{7}\left(B_{3}\left(n\right)\right)|, \end{array}$$

We obtain the following result after calculating it

$$⟹ReZG_{1}\left(B_{3}\left(n\right)\right)=\frac{2250}{11}n^{2}+\frac{27471}{143}n+\frac{15}{7}.$$

The $ReZG_{2}$ can be calculated by using (5) as follows

$$\begin{array}{ccc} ReZG_{2}\left(B_{3}\left(n\right)\right) & = & \frac{7}{10}|E_{1}\left(B_{3}\left(n\right)\right)|+\frac{2}{3}|E_{2}\left(B_{3}\left(n\right)\right)|+\frac{8}{15}\left|E_{3}\left(B_{3}\left(n\right)\right)\right|\\ & & +\frac{11}{24}|E_{4}\left(B_{3}\left(n\right)\right)|+\frac{2}{5}|E_{5}\left(B_{3}\left(n\right)\right)|+\frac{13}{40}\left|E_{6}\left(B_{3}\left(n\right)\right)\right|\\ & & +\frac{1}{4}\left|E_{7}\left(B_{3}\left(n\right)\right)\right|, \end{array}$$

We obtain the following result after calculating it

$$⟹ReZG_{2}\left(B_{3}\left(n\right)\right)=45n^{2}+51n+6.$$

The $ReZG_{3}$ index can be calculated from (6) as follows

$$\begin{array}{ccc} ReZG_{3}\left(B_{3}\left(n\right)\right) & = & 70|E_{1}\left(B_{3}\left(n\right)\right)\left|+54\right|E_{2}\left(B_{3}\left(n\right)\right)\left|+120\right|E_{3}\left(B_{3}\left(n\right)\right)|\\ & & +1024|E_{4}\left(B_{3}\left(n\right)\right)\left|+264\right|E_{5}\left(B_{3}\left(n\right)\right)\left|+250\right|E_{6}\left(B_{3}\left(n\right)\right)|\\ & & +1024|E_{7}\left(B_{3}\left(n\right)\right)|, \end{array}$$

We obtain the following result after calculating it

$$⟹ReZG_{3}\left(B_{3}\left(n\right)\right)=29880n^{2}+17232-660.$$

□

Now, we find $ABC_{4}$ and $GA_{5}$ indices of benzenoid hex planar octahedron network BHPOH(n).

**Theorem** **12.** *Let $B_{3}\left(n\right)$ be the benzenoid hex planar octahedron network. Then:*

- *
$ABC_{4}\left(B_{3}\left(n\right)\right)=\frac{27}{11}\sqrt{\frac{430}{19}}n^{3}+\left(\frac{36}{\sqrt{11}}-\frac{45\sqrt{8170}}{209}+\frac{18\sqrt{26}}{7}\right)+(60\sqrt{\frac{10}{209}}+6\sqrt{\frac{46}{77}}+6\sqrt{2}+\frac{36\sqrt{5}}{11}\;-\frac{12}{\sqrt{11}}+\frac{18\sqrt{8170}}{209}+\frac{3\sqrt{26}}{7})n+\left(24\sqrt{\frac{10}{77}}+6\sqrt{\frac{2}{7}}-6\sqrt{2}+2\sqrt{\frac{174}{35}}-3\frac{\sqrt{46}}{4}+\sqrt{\frac{86}{7}}\right);$
*
- *
$GA_{5}\left(B_{3}\left(n\right)\right)=\frac{18}{11}\sqrt{\frac{3}{19}}n^{3}+\left(\frac{36}{7}+18\sqrt{\frac{2}{77}}-\frac{30\sqrt{57}}{209}\right)+\left(\frac{395}{77}+6\sqrt{\frac{2}{209}}+\frac{12\sqrt{57}}{209}+4\sqrt{\frac{6}{11}}+\;\frac{24}{\sqrt{133}}\right)n+\left(\frac{3}{2}+16\sqrt{\frac{3}{77}}+4\sqrt{\frac{6}{133}}+4\sqrt{\frac{6}{35}}+2\sqrt{\frac{2}{7}}-4\sqrt{\frac{6}{11}}\right).$
*

**Proof.**  Using the edge partition, we have

$$\begin{array}{ccc} ABC_{4}\left(B_{3}\left(n\right)\right) & = & \frac{\sqrt{6090}}{210}|E_{1}\left(B_{3}\left(n\right)\right)|+\frac{2\sqrt{5}}{11}|E_{2}\left(B_{3}\left(n\right)\right)|+\frac{\sqrt{3542}}{154}\left|E_{3}\left(B_{3}\left(n\right)\right)\right|\\ & & +\frac{\sqrt{26}}{14}|E_{4}\left(B_{3}\left(n\right)\right)|+\frac{5\sqrt{266}}{266}|E_{5}\left(B_{3}\left(n\right)\right)|+\frac{1}{\sqrt{11}}\left|E_{6}\left(B_{3}\left(n\right)\right)\right|\\ & & +\frac{\sqrt{770}}{77}|E_{7}\left(B_{3}\left(n\right)\right)|+\frac{\sqrt{602}}{84}|E_{8}\left(B_{3}\left(n\right)\right)|+\frac{1}{\sqrt{14}}\left|E_{9}\left(B_{3}\left(n\right)\right)\right|\\ & & +\frac{\sqrt{2}}{4}|E_{10}\left(B_{3}\left(n\right)\right)|+\frac{\sqrt{\sqrt{46}}}{24}|E_{11}\left(B_{3}\left(n\right)\right)|+\frac{\sqrt{95}}{38}\left|E_{12}\left(B_{3}\left(n\right)\right)\right|\\ & & +\frac{\sqrt{2090}}{209}|E_{13}\left(B_{3}\left(n\right)\right)|+\frac{\sqrt{86}}{44}\left|E_{14}\left(B_{3}\left(n\right)\right)\right|, \end{array}$$

We obtain the following result by using Table 6

$$\begin{array}{ccc} ABC_{4}\left(B_{3}\left(n\right)\right) & = & \frac{27}{11}\sqrt{\frac{430}{19}}n^{3}+\left(\frac{36}{\sqrt{11}}-\frac{45\sqrt{8170}}{209}+\frac{18\sqrt{26}}{7}\right)+(60\sqrt{\frac{10}{209}}\\ & & +6\sqrt{\frac{46}{77}}+6\sqrt{2}+\frac{36\sqrt{5}}{11}-\frac{12}{\sqrt{11}}+\frac{18\sqrt{8170}}{209}+\frac{3\sqrt{26}}{7})n\\ & & +\left(24\sqrt{\frac{10}{77}}+6\sqrt{\frac{2}{7}}-6\sqrt{2}+2\sqrt{\frac{174}{35}}-3\frac{\sqrt{46}}{4}+\sqrt{\frac{86}{7}}\right). \end{array}$$

The index $GA_{5}$ can be determined from (8) as follows

$$\begin{array}{ccc} GA_{5}\left(B_{3}\left(n\right)\right) & = & \frac{2\sqrt{210}}{21}|E_{1}\left(B_{3}\left(n\right)\right)\left|+\right|E_{2}\left(B_{3}\left(n\right)\right)|+\frac{2\sqrt{154}}{25}\left|E_{3}\left(B_{3}\left(n\right)\right)\right|+\\ & & |E_{4}\left(B_{3}\left(n\right)\right)|+\frac{\sqrt{133}}{13}|E_{5}\left(B_{3}\left(n\right)\right)|+\frac{2\sqrt{154}}{29}\left|E_{6}\left(B_{3}\left(n\right)\right)\right|+\\ & & \frac{\sqrt{231}}{16}|E_{7}\left(B_{3}\left(n\right)\right)|+\frac{4\sqrt{14}}{15}|E_{8}\left(B_{3}\left(n\right)\right)|+\frac{4\sqrt{55}}{31}\left|E_{9}\left(B_{3}\left(n\right)\right)\right|\\ & & +\frac{2\sqrt{798}}{59}|E_{10}\left(B_{3}\left(n\right)\right)\left|+\right|E_{11}\left(B_{3}\left(n\right)\right)|+\frac{4\sqrt{57}}{31}\left|E_{12}\left(B_{3}\left(n\right)\right)\right|+\\ & & \frac{2\sqrt{418}}{41}|E_{13}\left(B_{3}\left(n\right)\right)\left|+\right|E_{14}\left(B_{3}\left(n\right)\right)|, \end{array}$$

We obtain the following result using Table 6

$$\begin{array}{ccc} GA_{5}\left(B_{3}\left(n\right)\right) & = & \frac{18}{11}\sqrt{\frac{3}{19}}n^{3}+\left(\frac{36}{7}+18\sqrt{\frac{2}{77}}-\frac{30\sqrt{57}}{209}\right)n^{2}+(\frac{395}{77}\\ & & +6\sqrt{\frac{2}{209}}+\frac{12\sqrt{57}}{209}+4\sqrt{\frac{6}{11}}+\frac{24}{\sqrt{133}})n+(\frac{3}{2}\\ & & +16\sqrt{\frac{3}{77}}+4\sqrt{\frac{6}{133}}+4\sqrt{\frac{6}{35}}+2\sqrt{\frac{2}{7}}-4\sqrt{\frac{6}{11}}). \end{array}$$

□

## 4. Comparison of Indices through Graphs

**Figure 4:**
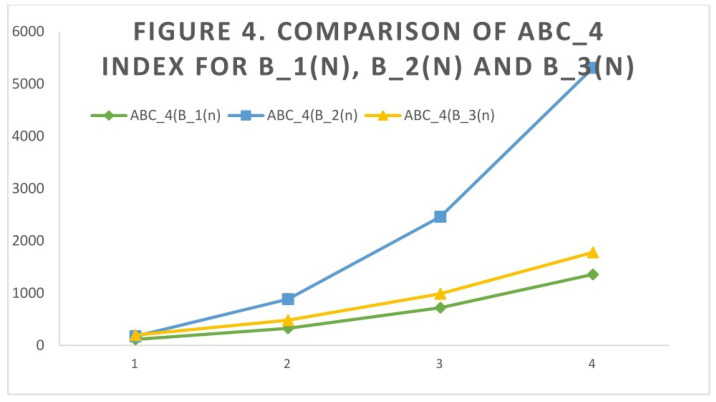
Comparison of $ABC_{4}$ index for $B_{1}\left(n\right)$, $B_{2}\left(n\right)$, and $B_{3}\left(n\right)$.

**Figure 5:**
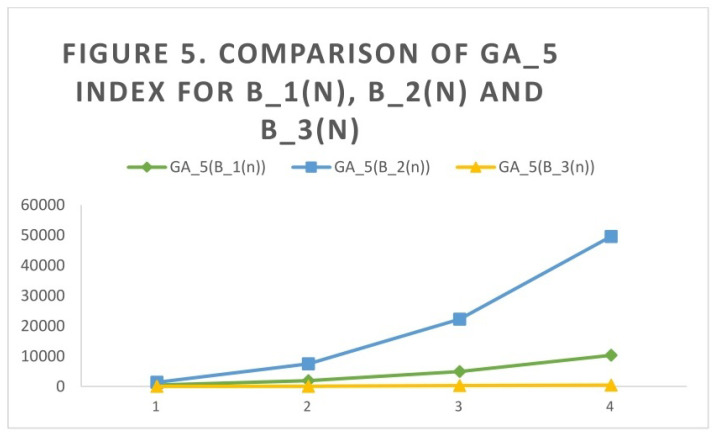
Comparison of $GA_{5}$ index for $B_{1}\left(n\right)$, $B_{2}\left(n\right)$, and $B_{3}\left(n\right)$.

The comparison of the of $ABC_{4}$ index and $GA_{5}$ index for $B_{1}\left(n\right)$, $B_{2}\left(n\right)$, and $B_{3}\left(n\right)$ is conducted for different values of *n*. The comparison graphs are shown in Figure 4 and Figure 5.

## 5. Applications

Graph theory is fast becoming a popular topic in mathematics because of its numerous applications in fields as varied as biochemistry (genomics), electrical engineering (communications networks and coding theory), computer science (algorithms and computations), and operations research. These results are also very useful for chemists who are working on such graphs.

## 6. Conclusions

The study of topological descriptors can help us construct basic network topologies. The specific result for the forgotten index, Balaban index, reclassified Zagreb indices, $ABC_{4}$ index, and $GA_{5}$ index of the benzenoid networks of type are contained in this study. Benzenoid networks have been researched in respect to several graph-ideological factors due to their fascinating and complicated characteristics. These results could be useful for computer scientists and chemists who deal with benzenoid networks.

## Figures and Tables

**Table 1:** Degree-based edge partition for BPOH(n).

(δ(s),δ(t))	Number of Edges	(δ(s),δ(t))	Number of Edges
(3,3)	$36n^{2}$	(4,8)	12n
(3,4)	12n	(8,8)	$18n^{2}-12n$
(3,8)	$36n^{2}-12n$		

**Table 2:** Edge partition based on sum of degrees for BPOH(n).

(δ(s),δ(t))	Number of Edges	(δ(s),δ(t))	Number of Edges
(10,10)	6n	(22,36)	12n
(10,14)	12n	(22,40)	12(n−1)
(14,14)	$36n^{2}-18n$	(36,40)	12
(14,36)	24	(40,40)	12n−18
(14,40)	48(n−1)	(40,44)	12(n−1)
(14,44)	$36n^{2}-60n+24$	(44,44)	$18n^{2}-36n+18$
(22,10)	12n		

**Table 4:** Edge partition based on sum of degrees for BDPOH(n).

(δ(s),δ(t))	Number of Edges	(δ(s),δ(t))	Number of Edges
(10,10)	12n−6	(22,36)	12n
(10,14)	24n−12	(22,40)	12(n−1)
(14,14)	$102n^{2}-114n+18$	(36,40)	12(n−1)
(14,36)	24n	(36,36)	6
(14,40)	24n	(40,44)	24(n−1)
(14,44)	$114n^{2}-210n+48$	(44,44)	$54n^{2}-114n+6$
(10,22)	24n−12		

**Table 6:** Edge partition based on sum of degrees for BHPOH(n).

(δ(s),δ(t))	Number of Edges	(δ(s),δ(t))	Number of Edges
(10,21)	12	(21,24)	12
(11,11)	18n	(21,38)	12n
(11,14)	12n	(24,11)	24n−24
(14,14)	$36n^{2}+6n$	(24,24)	12n−18
(14,38)	24n	(24,38)	12n−12
(21,11)	24n	(44,44)	$18n^{2}-12n$

## Data Availability

Not applicable.

## References

[1] Ali H., Siddiqui H.M.A., Shafiq M.K. (2016). On degree-based topological descriptors of oxide and silicate molecular structures. MAGNT Res. Rep..

[2] Randić M. (1975). Characterization of molecular branching. J. Am. Chem. Soc..

[3] Furtula B., Gutman I. (2015). A forgotten topological index. J. Math. Chem..

[4] Balaban A.T. (1982). Highly discriminating distance-based topological index. Chem. Phys. Lett..

[5] Balaban A.T., Quintas L.V. (1983). The smallest graphs, trees, and 4-trees with degenerate topological index. J. Math. Chem..

[6] Ranjini P.S., Lokesha V., Usha A. (2013). Relation between phenylene and hexagonal squeeze using harmonic index. Int. J. Graph Theory.

[7] Ghorbani M., Hosseinzadeh M.A. (2010). Computing ABC4 index of nanostar dendrimers. Optoelectron. Adv. Mater. Rapid Commun..

[8] Graovac A., Ghorbani M., Hosseinzadeh M.A. (2011). Computing fifth geometric-arithmetic index for nanostar dendrimers. J. Math. Nanosci..

[9] Ali H., Binyamin M.A., Shafiq M.K., Gao W. (2019). On the degree-based topological indices of some derived networks. Mathematics.

[10] Ali H., Sajjad A. (2019). On further results of hex derived networks. Open J. Discret. Appl. Math..

[11] Azari M. (2021). Multiplicative-sum Zagreb index of splice, bridge, and bridge-cycle graphs. Bol. Soc. Paran. Mat..

[12] Azari M., Iranmanesh A., Tehranian A. (2013). Two topological indices of three chemical structures. MATCH Commun. Math. Comput. Chem..

[13] Falahati-Nezhad F., Azari M. (2016). The inverse sum indeg index of some nanotubes. Studia Univ. Babes Bolyai Chem..

[14] Liu J.B., Bao Y., Zheng W.T., Hayat S. (2021). Network coherence analysis on a family of nested weighted n-polygon networks. Fractals.

[15] Liu J.B., Wang C., Wang S., Wei B. (2019). Zagreb indices and multiplicative Zagreb indices of eulerian graphs. Bull. Malays. Math. Sci. Soc..

[16] Liu J.B., Zhao J., Cai Z.Q. (2020). On the generalized adjacency, Laplacian and signless Laplacian spectra of the weighted edge corona networks. Phys. A Stat. Mech. Its Appl..

[17] Liu J.B., Zhao J., He H., Shao Z. (2019). Valency-based topological descriptors and structural property of the generalized sierpiński networks. J. Stat. Phys..

[18] Liu J.B., Zhang T., Wang Y., Lin W. (2022). The Kirchhoff index and spanning trees of Möbius/cylinder octagonal chain. Discret. Appl. Math..

[19] Nikolić S., Trinajstić N. (1995). The Wiener index: Development and applications. Croat. Chem. Acta.

[20] Wiener H. (1947). Structural determination of paraffin boiling points. J. Am. Chem. Soc..

